# A hierarchical approach in the diagnostic workflow of chronic myelomonocytic leukemia: Pivotal role of the “Mono‐dysplasia‐score” combined with flow cytometric quantification of monocyte subsets

**DOI:** 10.1111/ijlh.13115

**Published:** 2019-10-24

**Authors:** Jaja Zhu, Elise Sourdeau, Honorine Aubert, Sylvain Clauser, Agathe Maillon, Claude Capron, Katayoun Jondeau, Emily Ronez, Francoise Schillinger, Francine Garnache‐Ottou, Edouard Cornet, Valérie Bardet

**Affiliations:** ^1^ Service d'Hématologie‐Immunologie‐Transfusion Hôpitaux Universitaires Paris Ile De France Ouest Université Versailles Saint Quentin Boulogne France; ^2^ Laboratoire d'Hématologie UNICAEN CHU de Caen Normandie Normandie University Caen France; ^3^ Unité fonctionnelle d'Hématologie Service de Médecine Interne Hôpitaux Universitaires Paris Ile De France Ouest Université Versailles Saint Quentin Boulogne France; ^4^ Laboratoire d'Hématologie Etablissement Français du Sang de Bourgogne/Franche‐Comté Besançon France; ^5^ Laboratoire d'Hématologie UNICAEN CHU de Caen Normandie INSERM U1245 Normandie University Caen France

**Keywords:** chronic myelomonocytic leukemia, classical monocytes fraction, flow cytometry, monocyte subsets, mono‐dysplasia score

## Abstract

**Introduction:**

Monocytosis is a frequent trigger for blood smear review in a routine hematology laboratory whereas chronic myelomonocytic leukemia (CMML) is infrequent and arises mostly in elderly patients. In order to define the best workflow for monocytosis, we studied three diagnostic approaches: the classical morphology approach (blood smear review), the flow cytometry assay (quantification of monocyte subsets as described by Selimoglu‐Buet *et al* in 2015), and the “mono‐dysplasia‐score” also referred to as “Monoscore (as described by our team in 2018 using the structural parameters of the Sysmex XN™ analyzers).

**Methods:**

Studying a multicentric cohort of 196 nonclonal monocytoses and CMML patients aged over 50 years, we compared the diagnostic performance of the three approaches alone and in combination to propose a diagnostic decision tree.

**Results:**

In patients presenting with additional criteria for slide review to monocytosis (37% of our cohort), we propose to sequentially combine morphology, Monoscore, and flow cytometry. On the contrary, for patients with isolated monocytosis (63%), slide review is not mandatory and we suggest performing flow cytometry depending on the Monoscore value. Using the proposed algorithm, 98% of CMML patients would have been correctly identified, slide review rate drastically reduced, and flow cytometry would have been carried out in 44% of patients.

**Conclusion:**

We have shown that implementation of Monoscore is a useful input filter to significantly reduce slide reviews without losing sensitivity and that flow cytometry is a performant technique in the second step of the diagnostic workup of CMML.

## 1 INTRODUCTION

According to the updated World Health Organization (WHO) classification of Tumors of Haematopoietic and Lymphoid Tissues,^1^ the diagnosis of chronic myelomonocytic leukemia (CMML)^2^ requires both positive criteria: the presence of persistent peripheral monocytosis (≥1 × 10^9^/L) and monocytes accounting for ≥10% of the total white blood cell (WBC) count, and negative criteria: absence of WHO criteria for myeloproliferative neoplasms or acute myeloid leukemia, and absence of disease‐defining translocations. Finally, CMML diagnosis requires identification of myelodysplasia or acquired clonal cytogenetic or molecular genetic abnormality or exclusion of nonclonal monocytosis. CMML combines myeloid cell proliferation with myeloid cell dysplasia and has thus been classified by the WHO as a myelodysplastic syndrome/myeloproliferative neoplasm (MDS/MPN).^1^ The WHO recognizes two prognostic parameters: WBC count and blast percentage. A WBC count ≥13 × 10^9^/L separates MPN‐CMML, in which the RAS/MAPK signaling pathway is frequently activated, from MDS‐CMML, in which prognosis is better.^2^, ^3^ Blast cell percentage separates patients into 3 groups: CMML‐0 (<2% blasts in peripheral blood (PB) and <5% in bone marrow (BM)), CMML‐1 (2%‐4% in PB and/or 5%‐9% in BM), and CMML‐2 (5%‐19% blasts in PB and/or 10%‐19% in BM and/or Auer rods are present). The distinction between promonocytes, which must be counted as blast cells, dysplastic monocytes, and dysplastic granulocytes requires expertise.^4^ Myelodysplasia can be minimal or absent in some cases, especially in CMML‐0, cytogenetic abnormalities are detected only in ~30% of patients^5^ and a clonal abnormality in >90% of cases.^3^, ^6^ In these elderly patients (median age at CMML diagnosis ~ 71‐74 years), genetic findings must be interpreted with caution due to the frequency of age‐related mutations^7^ and the presence of some of these mutations in other neoplasms. A recent paper showed a diagnostic signature typical of CMML using the quantification of monocyte subsets by flow cytometry.^8^ In healthy conditions, the majority of monocytes (85 ± 6%) are classical monocytes (cMo, CD14^++^ CD16^−^), accompanied by intermediate monocytes (iMo, CD14^++^ CD16^+^, 5 ± 2%) and nonclassical monocytes (ncMo, CD14^low/neg^ CD16^++^, 10 ± 2%).^9^, ^10^ During the course of an infection or in inflammatory conditions, there is an increase first of intermediate monocytes, followed by nonclassical monocytes.^10^ CMML is characterized by an increase in the fraction of classical monocytes ≥94% at the expense of intermediate and nonclassical monocytes, whereas nonclonal monocytosis is due to the accumulation of intermediate and nonclassical monocytes.^8^ This flow cytometry assay has a good sensitivity (93.6%) and specificity (89.7%) and can be implemented in multiple diagnostic laboratories with an excellent correlation in the classical monocyte fraction measurement.^11^, ^12^ Monocytosis is a frequent abnormality on a complete blood count (CBC) and differential, and strictly following the WHO recommendations (ie, reviewing the blood smear for signs of myelodysplasia or excess blasts if the WHO criteria for CMML are met) would generate a microscopic blood smear review in around 6%‐7% of samples (6.5% in our experience). Considering the low frequency of CMML (0.4 cases per 100 000 population),^2^ the International Society for Laboratory Hematology^13^ and the Groupe Francophone d'Hématologie Cellulaire (GFHC) 14 both recommended a blood smear review if the monocyte count exceeds 1.5 × 10^9^/L on the first CBC or if monocytosis persists for more than 30 days in adult patients to avoid an excess of unnecessary smear reviews, reducing the rate to 1.2%.^15^ In a recent study,^16^ using structural parameters of the Sysmex XN™ analyzers, we described the “mono‐dysplasia‐score” also referred to as “Monoscore.” This score incorporated three parameters: neutrophil/monocyte ratio, structural neutrophil dispersion (Ne‐WX), and monocyte absolute count and was calculated as soon as the WHO criteria were met (monocyte count ≥ 1 × 10^9^/L and monocytes accounting for ≥10% of the WBC count). Structural neutrophil dispersion is increased in the presence of hypogranulated/degranulated neutrophils, a hallmark of dysplasia in the context of CMML, while the neutrophil/monocyte ratio shows evidence of monocytic proliferation outside of an infectious context. Contrary to morphology, this approach is fully standardized, operator independent, and immediate. High sensitivity (92.3%) and specificity (93.6%) of the Monoscore were confirmed in a validation cohort of more than 1800 unselected samples of adult patients with monocytosis according to the WHO criteria. Due to its high negative predictive value (NPV, 99.7% and 99.9% in the learning and validation cohorts, respectively), using Monoscore to trigger blood smear review lowered the review rate from 6%‐7% to 0.5% without losing sensitivity. In the present multicentric study, we investigated and compared the diagnostic performance of the classical morphology approach (blood smear review) to the flow cytometry assay and the Monoscore in order to define the best workflow for investigating monocytosis in routine hematology laboratories.

## 2 MATERIALS AND METHODS

### 2.1 Patients

As described in the flowchart (Figure S1), 271 patients falling within the WHO criteria (monocyte count ≥ 1 × 10^9^/L and accounting for ≥10% of the total WBC) were included in this multicentric (Paris, Besançon and Caen) French study between December 2015 and December 2017. Patients younger than age 50 (n = 35) were excluded from this study due to the very low frequency of CMML in these patients. Patients with overt context of nonclonal monocytosis (active sepsis, n = 25), ineligible for flow cytometry (sample age >24 hours, recovery from aplasia, n = 11) or without follow‐up (n = 4) were excluded, leaving 196 samples for analysis (Figure S1). C‐reactive protein (CRP) was measured for 132 of 196 patients on Vitros 5600 (Ortho Clinical Diagnostics, Issy les Moulineaux, France) and AU680 (Beckman Coulter) analyzers (Table 1).

**Table 1:** Univariate analysis of basic characteristics of the study population

	Nonclonal monocytosis, n = 136	CMML, n = 60	*P*‐value
Parameter (median, range)			
Age, years (range)	76 (51‐103)	84 (54‐104)	.001
Gender (male/female)	80/56	29/31	.23
Besançon	16 (12%)	12 (20%)	
Caen	65 (48%)	14 (23%)	
Paris	55 (40%)	34 (57%)	
WBC (10^9^/L)	9.2 (3.7‐25.0)	9.8 (4.3‐303.6)	.14
Hemoglobin (g/dL)	11.3 (7.3‐16.2)	10.7 (5.2‐15.7)	.16
Platelets (10^9^/L)	222 (29‐810)	140 (10‐781)	<.0001
Neutrophils (10^9^/L)	5.3 (0.3‐19.2)	4.7 (0.3‐157.9)	.90
Immature granulocytes (%)	1.0 (0.0‐18.0)	1.7 (0.0‐29.0)	.001
Immature granulocytes (10^9^/L)	0.1 (0.0‐2.5)	0.2 (0.0‐51.8)	.002
Blast cells (%)	0 (0.0‐5.0)	0 (0.0‐6.5)	.29
Monocytes (%)	17.3 (10.1‐57.0)	26.1 (12.6‐57.6)	<.0001
Monocytes (10^9^/L)	1.6 (1.0‐6.3)	2.6 (1.2‐82.0)	<.0001
C‐reactive protein (mg/L), n = 132	26.0 (0‐361)	16.5 (1.4‐296)	.10
Blood smear dysplasia score	0 (0‐2)	1 (0‐4)	<.0001
Monoscore	0.15 (0.00‐1.00)	0.98 (0.07‐1.00)	<.0001
Classical monocytes fraction	90.0 (16.3‐99.4)	97.0 (83.3‐100.0)	<.0001
Blood smear indications additional to monocytosis (n, %)			
Analyzer flag	19 (14%)	28 (47%)	<.0001
Platelets < 100 × 10^9^/L	21 (15%)	18 (30%)	.03
Immature granulocytes ≥ 10%	6 (4%)	9 (15%)	.02

This study is a noninterventional research study, all investigations performed in this study were approved by the local Institutional Review Board (Comité d'Ethique Recherche Paris‐Saclay‐Polethis: 2019‐08291) and the procedures followed were in accordance with the Helsinki Declaration.

### 2.2 Complete blood count and blood smear examination

All samples were collected in EDTA 3K or EDTA 2K tubes from Becton Dickinson^TM^ and analyzed on a Sysmex XN‐10 analyzer (Sysmex) within 6 hours after blood collection, with smears prepared accordingly by an SP‐10 slidemaker/stainer (Sysmex) at the same time. Blood smears were analyzed for all patients, the quantitative or qualitative (analyzer flag) abnormalities on the CBC or differential justifying the realization of the smear (in addition to monocytosis itself) are detailed in Table S1. As specific recommendations for CMML diagnosis and evaluation of dysplasia on blood smears are not available, two hundred cells (leukocytes and platelets) were analyzed and the cutoff for significant dysplasia was arbitrarily set at 10% of each lineage, following WHO recommendations for the diagnosis of myelodysplastic syndromes.^17^ Blood smear dysplasia was quantified by well‐trained morphologists (hematologists with more than 10 years’ experience) blind to the consensus diagnosis and reported as a global score according to the criteria mentioned in Table S1 in order to standardize the data.

### 2.3 Monoscore

Monoscore was automatically calculated for all samples falling within the WHO criteria. No intersite reanalysis was carried out since the previous Schillinger et al^16^ study already demonstrated that the score performance in such conditions was not analyzer‐dependent. As previously published, a Monoscore ≥0.161 was considered as an abnormal result suggesting increased probability of CMML.

### 2.4 Flow cytometry

Quantification of monocyte subsets was performed as previously reported^8^ on the same EDTA sample within 24 hours following collection. All samples were immunophenotyped according to local procedures using 6‐color immunophenotyping panels on Navios (Beckman Coulter) or Canto II (BD Biosciences) flow cytometers. Details of the clones and fluorochromes used are given in Table S2. Multicentric blind reanalysis of 61 cytometry files collected from the three laboratories demonstrated an excellent correlation in classical monocyte fraction measurement (*r* = .962; *P* < 10^−4^) as previously published,^11^ and full agreement was obtained in all samples with a cutoff value of 94% classical monocytes, also referred to as the “cMo fraction,” as the critical readout value.

Consensus diagnosis (CMML or nonclonal monocytosis) was established by combining blood cell morphology, bone marrow examination, cytogenetics, molecular analyses if available and 3‐month follow‐up according to the WHO criteria.^2^ Consensus diagnosis was blinded to flow cytometry quantification of monocyte subsets and Monoscore value. After follow‐up, CMML diagnosis was achieved in 60 patients and nonclonal monocytosis was confirmed for 136. The clinical and biological characteristics of the study population are reported in Table 1.

### 2.5 Statistics

Categorical and continuous variables are reported as numbers and percentages, or median and range (min‐max), respectively. Association between CMML diagnosis and categorical and continuous parameters was evaluated using a chi‐square test and Mann‐Whitney test, respectively. Multivariate analysis, including all variables with *P* < .05, was then performed with logistic regression. The diagnostic performance of morphology (blood smear review), Monoscore, flow cytometry quantification of monocyte subsets was assessed as an individual method for checking CMML diagnosis by nonparametric receiver‐operator curve (ROC) analysis. Sensitivity, specificity, positive predictive value (PPV), and negative predictive value (NPV) were computed with 95% confidence intervals and calculated from the published cutoff values for all methods.

A regression tree, based on the CHAID algorithm, was then established including published score thresholds to define the most efficient workflow. The only limitation was that a smear review was done when imposed by other criteria (analyzer flag, abnormal results leading to a smear review as per GFHC recommendations).

All the tests were two‐sided at a significance level of *P* < .05. All analyses were performed using R statistical software V.3.3.2 (R Foundation for Statistical Computing).

## 3 RESULTS

### 3.1 Characteristics of the study population

The studied population included 136 patients presenting with nonclonal monocytosis (controls) and 60 patients with CMML. Univariate analysis of baseline characteristics of the cohort revealed eight parameters associated with CMML diagnosis (Table 1). As expected, CMML patients were significantly older than nonclonal monocytosis patients (84 [54‐104] versus 76 years [51‐103], *P* = .001) (Table 1), showed a higher monocyte count (2.6 × 10^9^/L [1.2‐82.0] versus 1.6 × 10^9^/L [1.0‐6.3], *P* < 10^−4^) and a lower platelet count (140 × 10^9^/L [10‐781] versus 222 × 10^9^/L [29‐810], *P* < 10^−4^). Immature granulocyte percentage was also slightly higher in CMML patients (1.7% [0.0‐29.0] versus 1.0% [0.0‐18.0], *P* = .001) whereas WBC count, neutrophil count, blast percentage, and other CBC parameters were not significantly different. The Monoscore value was significantly higher in CMML patients than in controls (0.98 [0.07‐1.000] versus 0.15 [0.000‐1.000], *P* < 10^−4^). Consistent with the observation that inflammation is a frequent hallmark of CMML,^18^ CRP levels were not different between CMML and controls (16.5 [1‐296] versus 26 [0‐361], *P* = .10). As morphological analysis of a blood smear is the gold standard to decide for further explorations, we quantified and analyzed dysplasia in our cohort. As described earlier, we arbitrarily designed a score attributing 1 point for each significantly (≥10%) observed dysplasia (Table S1). This dysplasia score was significantly higher in CMML patients than in nonclonal monocytoses (1 [0‐4] versus 0 [0‐2], *P* < 10^−4^) (Table 1). As expected, the classical monocyte fraction measured by flow cytometry was also significantly higher in CMML patients than in controls (97.0 [83.3‐100.0] versus 90.0 [16.3‐99.4], *P* < 10^−4^). We next performed a multivariate logistic regression model considering all variables significantly associated with CMML diagnosis (*P* < .05), that is age, platelet count, immature granulocyte percentage (but not immature granulocyte absolute count for an obvious collinearity issue), blood smear dysplasia score, Monoscore (but not monocyte absolute count also for a collinearity issue), and classical monocyte fraction. Only Monoscore (ORR = 45.9, *P* = .0006), blood smear dysplasia score (ORR = 51.53, *P* < 10^−4^), and classical monocyte fraction (ORR = 1.70, *P* < 10^−4^) remained strong predictors of CMML diagnosis (Table 2).

**Table 2:** Association of study parameters with CMML diagnosis using multivariate analysis

Parameter	ORR	95% CI	*P*‐value
Age	1.02	0.94‐1.10	.62
Gender	1.30	0.27‐6.35	.74
Platelet count	1.00	0.99‐1.01	.85
Immature granulocytes (%)	1.17	0.96‐1.42	.12
Blood smear dysplasia score	51.53	8.71‐304.71	<.0001
Monoscore XN	45.9	5.16‐408.25	.0006
Classical monocyte fraction	1.70	1.33‐2.37	<.0001

### 3.2 Performance of a single diagnostic approach for CMML diagnosis

The performance of blood smear dysplasia score, Monoscore, and classical monocyte fraction as diagnostic markers was then tested. Blood smear dysplasia score ≥1 had a PPV of 75% (95% CI: 65 to −85) and a NPV of 94% (95% CI: 90‐98) (Figure S2A), while Monoscore ≥0.161 had a PPV of 46% (95% CI: 38‐55) and a NPV of 97% (95% CI: 93‐100) (Figure S2B), and classical monocyte fraction ≥94% a PPV of 71% (95% CI: 61‐81) and a NPV of 97% (95% CI: 93‐100) (Figure S2C). Interestingly, despite close NPVs for the three techniques, the two Monoscore false‐negative CMML patients had positive blood smear dysplasia score and one had increased classical monocyte fraction. Conversely, all eight false‐negative samples with the blood smear dysplasia score were correctly identified with both Monoscore and classical monocyte fraction quantification. Three CMML patients did not present with an increased classical monocyte fraction, but all of them harbored a typical bulbous aspect as described in CMML associated with inflammatory conditions.^11^ Two of them were detected both by Monoscore and blood smear dysplasia score and one patient was only detected by blood smear review. Thus, the three techniques appear to be complementary for an optimal CMML diagnosis.

### 3.3 Performance of combined diagnostic approaches for CMML diagnosis

To determine the best workflow in routine hematology laboratories, we compared the performance of the association of Monoscore plus blood smear dysplasia score, the blood smear dysplasia score plus classical monocyte fraction and Monoscore plus classical monocyte fraction. Monoscore ≥0.161 or blood smear dysplasia score ≥1 had a PPV of 45% (95% CI: 37‐54 and a NPV of 100% (95% CI: 100‐100) (Table 3), while blood smear dysplasia score ≥1 or classical monocyte fraction ≥94% had a PPV of 62% (95% CI: 52‐72) and a NPV of 100% (95% CI: 100‐100) (Table 3). Monoscore ≥0.161 or classical monocyte fraction ≥94% had a PPV of 43% (95% CI: 35‐51) and an NPV of 98% (95% CI: 95‐100) (Table 3). Absence of multicollinearity of the three methods was assessed by checking the variance inflation factor (VIF).

**Table 3:** Contingency table of combined techniques

Combination	nonclonal monocytosis (n = 136)	CMML (n = 60)
Monoscore ≥ 0.161 or blood smear dysplasia score ≥ 1	73	60
Monoscore < 0.161 and blood smear dysplasia score = 0	63	0
Monoscore ≥ 0.161 or cMo ≥ 94%	79	59
Monoscore < 0.161 and cMo < 94%	57	1*
Blood smear dysplasia score ≥ 1 or cMo ≥ 94%	37	60
Blood smear dysplasia score = 0 and cMo < 94%	99	0

* Patient with a typical “bulbous” aspect.

### 3.4 Hierarchy of the diagnostic approaches for CMML diagnosis: designing the best workflow for monocytosis

As the three techniques are not equivalent in terms of cost and time consumption, we analyzed the respective place of each in the workflow of monocytosis. As other criteria for slide review in addition to monocytosis may be present (Table 1), we first considered this. In our cohort, 72 patients (37%) with monocytosis had at least one other criterion for slide review, including 35 of 136 (26%) nonclonal monocytoses and 37 of 60 (62%) CMML. In these patients, blood smear morphology correctly identified 29 of 35 nonclonal monocytoses and 35 of 37 (95%) CMML patients. Monoscore was ≥0.161 in all CMML but one, which had a positive blood smear and presented a “bulbous” flow cytometry aspect (Table 4). Two CMML patients were negative for morphology and presented with both an abnormal Monoscore and increased nonclassical monocyte fraction.

**Table 4:** Contingency table of combined techniques relative to the presence or absence of an additional trigger to monocytosis for slide review

With another trigger for slide review	nonclonal monocytosis, n = 35		CMML, n = 37	
	n = 13		n = 1	
	cMo < 94%	cMo ≥ 94%	cMo < 94%	cMo 1 ≥ 94%
Monoscore < 0.161				
Blood smear dysplasia score ≥ 1	0	1	1*	0
Blood smear dysplasia score = 0	9	3	0	0
	n = 22		n = 36	
	cMo < 94%	cMo ≥ 94%	cMo < 94%	cMo ≥ 94%
Monoscore ≥ 0.161				
Blood smear dysplasia score ≥ 1	5	0	1*	33
Blood smear dysplasia score = 0	15	2	0	2

No other trigger for slide review	Nonclonal monocytosis, n = 101		CMML, n = 23	
	n = 56		n = 1	
	cMo < 94%	cMo ≥ 94%	cMo < 94%	cMo ≥ 94%
Monoscore < 0.161				
Blood smear dysplasia score ≥ 1	4	1	0	1
Blood smear dysplasia score = 0	44	7	0	0
	n = 45		n = 22	
	cMo < 94%	cMo ≥ 94%	cMo < 94%	cMo ≥ 94%
Monoscore ≥ 0.161				
Blood smear dysplasia score ≥ 1	5	1	2*	14
Blood smear dysplasia score = 0	31	8	0	6

* Patient with a typical “bulbous” aspect.

* Patients with a typical “bulbous” aspect.

A total of 124 patients (63%) did not present with an additional trigger for slide review other than monocytosis, including 101 of 136 (74%) nonclonal monocytoses and 23 of 60 (38%) CMML patients. In these patients, Monoscore was positive (>0.161) for 22 of 23 CMML, the remaining patient was correctly identified by both blood smear morphology and flow cytometry. Blood smear morphology correctly identified 90 of 101 (89%) nonclonal monocytoses and 17 of 23 (74%) CMML patients. Six CMML patients were thereby negative for morphology but all had an abnormal Monoscore and 4 of 6 had increased nonclassical monocyte fraction. The two remaining patients presented with a “bulbous” aspect by FCM due to concomitant inflammatory conditions. Flow cytometry was controlled a few months later in those patients with a “bulbous” aspect and showed a classical monocyte fraction ≥94%.

**Figure 1:**
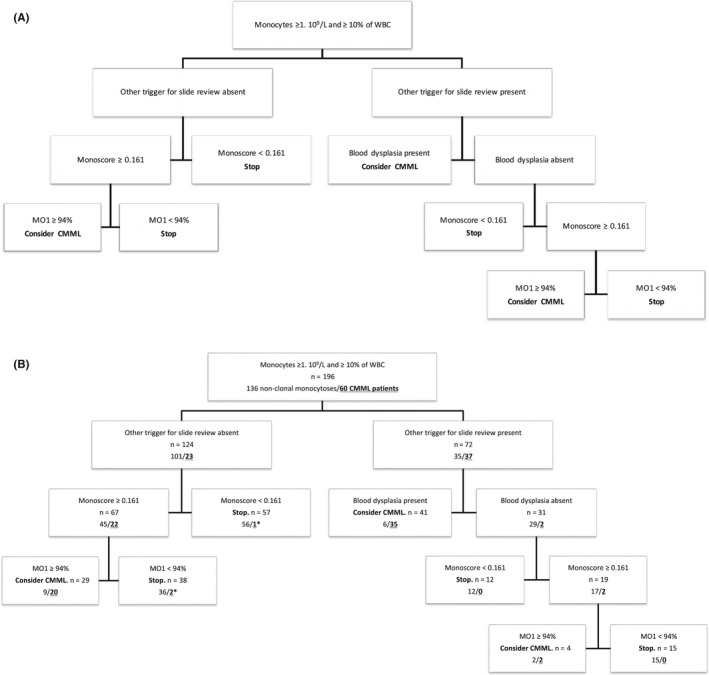
A, Decision tree in the diagnostic workflow of monocytosis. B, Application of the proposed algorithm to our cohort. Numbers of CMML patients are figured in bold and underlined numbers. For patients with another trigger for slide review than monocytosis (n = 72 including 37 CMML patients), significant blood smear dysplasia (n = 41) enables CMML diagnosis in most CMML cases (35 of 37). Patients with another trigger for slide review, no significant dysplasia on the blood smear but a Monoscore ≥ 0.161 (n = 19) might benefit from flow cytometric quantification of monocyte subsets to exclude or confirm (2 of 37) CMML diagnosis. Patients not presenting other criteria for slide review than monocytosis (n = 124) and with Monoscore < 0.161 (n = 57) do not need further exploration. Patients with Monoscore ≥ 0.161 (n = 67) would benefit from flow cytometric quantification of monocyte subsets which allows direct identification of 20 of 22 CMML patients plus 2 patients harboring inflammatory profiles. *Indicates CMML patients with an inflammatory “bulbous” flow cytometry profile

A regression tree, based on the CHAID algorithm, was then established including published score thresholds to define the most efficient workflow with the only limitation that a smear review was performed when imposed by other criteria than monocytosis. This algorithm is depicted in Figure 1A and the results of its application on our cohort in Figure 1B. For patients with other triggers for slide review (n = 72, including 37 CMML patients), blood smear examination allowed correct classification of 35 of 37 CMML patients and 29 of 35 nonclonal monocytoses. Monoscore correctly excluded 12 nonclonal monocytoses and was abnormal in the remaining 19 patients including 2 CMML patients. As the last step of the algorithm, flow cytometry correctly excluded 15 nonclonal monocytoses and identified the 2 remaining CMML patients. Patients not presenting other criteria for slide review than monocytosis (n = 124, including 23 CMML patients) were stratified according to the Monoscore value, 57 patients (including one CMML patient) had a Monoscore <0.161 and would not be subjected to any additional investigation according to our proposed algorithm. Sixty‐seven patients had an abnormal Monoscore, including 22 CMML patients, who were correctly identified by flow cytometry for 20 of 22, the remaining 2 patients harbored an inflammatory profile with a “bulbous” aspect, and flow cytometry confirmed CMML diagnosis when performed a few months later.

## 4 DISCUSSION

In this study, we compared the diagnostic performances of three approaches that are commonly available in hematology routine laboratories, two standardized and reproducible techniques, Monoscore and flow cytometry, and one more operator‐dependent technique, morphologic quantification of dysplasia. To limit the influence of the operator in the latter, all slides were analyzed by well‐trained morphologists and dysplasia was quantified using a blood smear dysplasia score as mentioned previously. All techniques demonstrated good performance with high sensitivities, the lowest being for morphology at 87% (Figure S2A). Conversely, specificities showed a wider variation, ranging from 51% for Monoscore to 83% and 88% for flow cytometry and morphology, respectively (Figure [Link], [Link], [Link]A‐C). Interestingly, the different techniques showed high complementarity, and combining Monoscore and morphology or morphology and flow cytometry increased the NPV to 100%, while combining the two non‐operator‐dependent techniques, Monoscore and flow cytometry, increased the NPV to 98% (Table 3). This slightly lower NPV for the latter combination is due to the frequent occurrence of inflammation in CMML patients. CMML is frequently associated with systemic inflammatory and autoimmune diseases like systemic vasculitis, connective tissue disease, inflammatory arthritis, or neutrophilic disorder.^18^ This CMML‐associated inflammation increases the number of intermediate monocytes and artificially normalizes the percentage of classical monocytes despite the decrease of nonclassical monocytes. Those patients harbor a characteristic “bulbous” aspect in flow cytometry due to the near disappearance of the nonclassical monocytes and increase of the intermediate fraction.^19^ In a majority of cases, another flow cytometric quantification of the monocyte subsets performed a few months later allows to diagnose CMML by showing the expected increase in the classical monocyte fraction. Nonclassical monocytes specifically express a carbohydrate modification of P‐selectin glycoprotein ligand‐1, 6‐Sulfo LacNAc, also referred to as the slan antigen whereas intermediate monocytes are slan‐negative.^20^ As recently proposed,^21^ use of an additional antislan antibody in the flow cytometry panel dedicated to monocytosis would allow easy identification of CMML patients despite inflammatory conditions being present and drastically increase the sensitivity and specificity of this technique.

From a more practical and economical point of view, the three techniques are not equivalent. Monoscore is operator independent, immediately available, and costless, whereas morphology is operator‐dependent, necessitates highly trained morphologists to obtain such a good performance as the one described in this study, and is time‐consuming more than directly costly. Flow cytometry, requiring specific equipment and costly reagents, is not available in every routine hematology laboratory. However, flow cytometry has a high diagnostic performance and despite being, strictly speaking, operator‐dependent, has demonstrated good reproducibility in most applications and specifically in the context of CMML diagnosis.^11^ We therefore analyzed the performance of several combinations of these techniques, to define the best workflow in a routine diagnostic strategy and as a conclusion of our work we propose the decision tree described in Figure 1A. In a routine hematology laboratory, exploration of monocytosis by strictly following the WHO recommendations generates a large amount of slide reviews, around 6%‐7% in a university hospital, and integration of the real frequency of CMML in this reasoning does not support a systematic slide review policy. The same restrictions apply to flow cytometry and it is hard to consider ruling a quantification of monocyte subsets for every monocytosis without other criteria. As one third of patients falling within the WHO criteria presented another trigger for slide review, we considered those patients separately. In patients presenting with additional criteria for slide review, in line with the results of our previous multicentric study, Monoscore has excellent sensitivity and only one CMML patient was false negative but correctly detected by morphology. Blood smear review allowed correct identification of a vast majority of CMML patients (94%) and flow cytometry does not seem to be mandatory in these cases to reach CMML diagnosis. Two CMML patients were not detected by morphology and we suggest performing flow cytometry in all patients with abnormal Monoscore and absence of significant blood smear dysplasia (Figure 1A) to reach optimal sensitivity in CMML diagnosis. On the contrary, for patients with the sole criterion of monocytosis for slide review, morphology demonstrated lower performance compared to flow cytometry, the latter identifying six CMML patients which were not detected by morphology (27%) (Table 4). We can hypothesize that such CMML patients without other trigger for slide review are patients with a less advanced disease and that morphology shows lower sensitivity in this context. Only one CMML patient was false negative with Monoscore: this patient had a proliferative form of CMML, isolated monocytosis, no cytopenia but a splenomegaly which would justify blood smear review regardless of the CBC. We can hypothesize that, being mostly based on the evaluation of dysplasia, Monoscore has better performance in identifying dysplastic CMML patients compared to proliferative CMML patients. Due to the higher WBC and the high frequency of splenomegaly in those patients, blood smear examination may be triggered by other indications (including clinician request). Due to the lower sensitivity of morphology in those patients with isolated monocytosis, who represent two thirds of our cohort, we strongly recommend in our decision algorithm (Figure 1A) to directly perform quantification of monocyte subsets for all patients with isolated monocytosis and abnormal Monoscore. Patients with isolated monocytosis and Monoscore <0.161 do not need further explorations considering the high NPV of Monoscore (Figure 1A). Blood smear review is therefore not mandatory in these patients (two thirds of our cohort) with isolated monocytosis and a significant number of slide reviews will be avoided. Using the proposed decision tree, 59 of 60 CMML patients would have been correctly identified (98%), 63% of slide reviews would not have been performed and flow cytometry would have been carried out in 44% of patients to identify CMML patients who represent 31% of this selected cohort.

Flow cytometry is not widely available/affordable compared to morphology; however, certain centers have replaced microscopic WBC differential by flow cytometric WBC differential using the HematoFlow strategy,^22^ illustrating the growing place of flow cytometry in widespread applications. In a recent paper,^23^ the HematoFlow solution demonstrated a good sensitivity for CMML screening but the classical monocyte fraction was overestimated with this approach which needed to be completed by the reference method.^8^

From an ethical and medico‐economical point of view, it is interesting to emphasize that the combination of Monoscore, blood morphology, and flow cytometry correctly identifies all CMML patients solely with blood‐based diagnostic approaches. Although bone marrow examination remains mandatory for CMML patients (bone marrow blast percentage, cytogenetics, molecular analyses…), a significant number of bone marrow examinations could perhaps be avoided in nonclonal monocytoses using such a combined strategy leading to pursue investigations only in selected patients, for instance those suspected to have a CMML diagnosis according to our decision tree (Figure 1A).

To conclude, studying a cohort of 136 nonclonal monocytoses and 60 CMML patients, we have shown that the implementation of Monoscore is a useful input filter to significantly reduce slide reviews without losing sensitivity in the workflow of monocytosis and that in patients with isolated monocytosis, flow cytometry has superior performance to morphology (blood smear quantification of dysplasia) in the diagnostic workup of CMML. These results need to be confirmed in larger cohorts and the place of the new flow cytometric quantification of monocyte subsets using the antislan antibody to be determined.

## CONFLICT OF INTEREST

The authors declare no conflict of interest.

## AUTHOR CONTRIBUTIONS

JZ, ES, HA, AM, CC and ER collected and analyzed the biological data, KJ collected the clinical data, SC, FS, FGO, and EC analyzed the data and wrote the paper, VB designed the study, analyzed the data, performed the statistical analysis, and wrote the paper.
