# Supplementary figures and images for: A hierarchical approach in the diagnostic workflow of chronic myelomonocytic leukemia: Pivotal role of the “Mono‐dysplasia‐score” combined with flow cytometric quantification of monocyte subsets
Source: Int J Lab Hematol. 2019 Oct 24;41(6):782–90. doi: 10.1111/ijlh.13115 (PMC6916376; doi:10.1111/ijlh.13115)

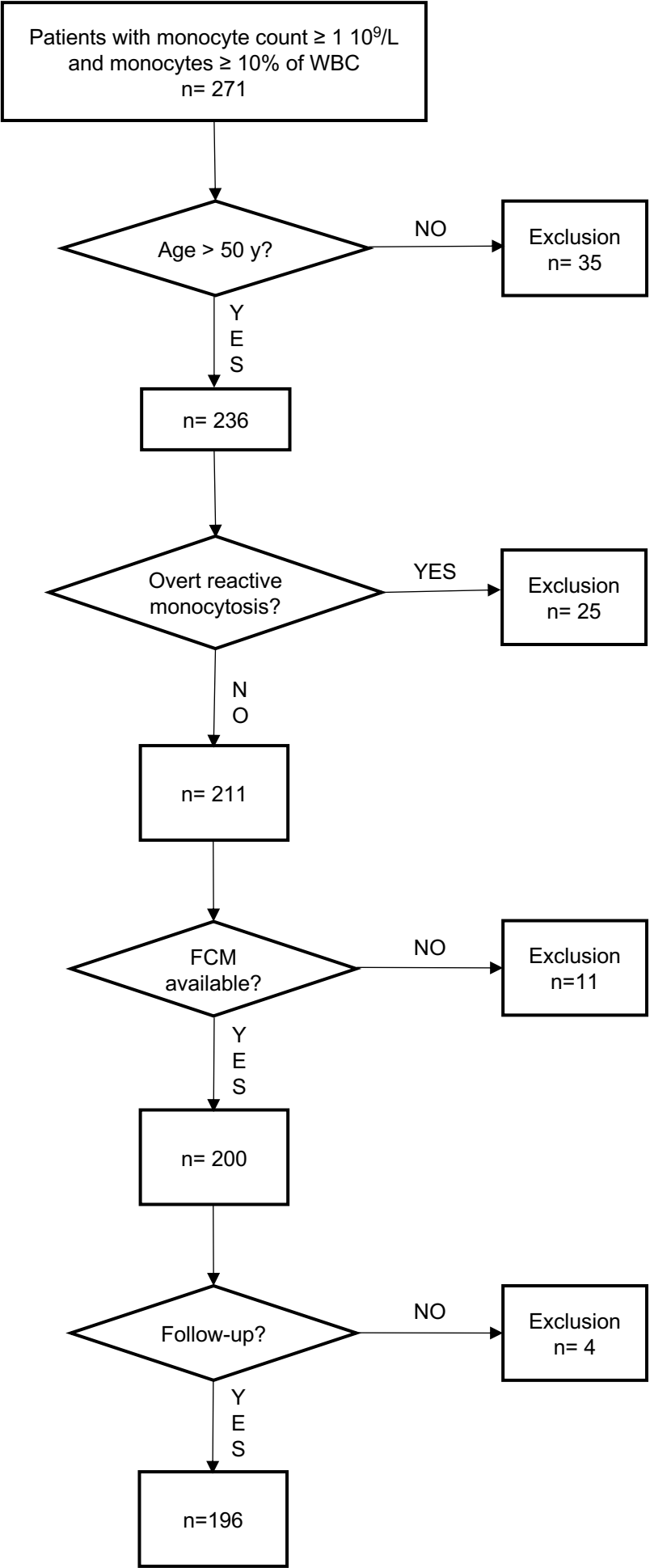

Supplement: Supplementary file 1 [file IJLH-41-782-s001.pdf]

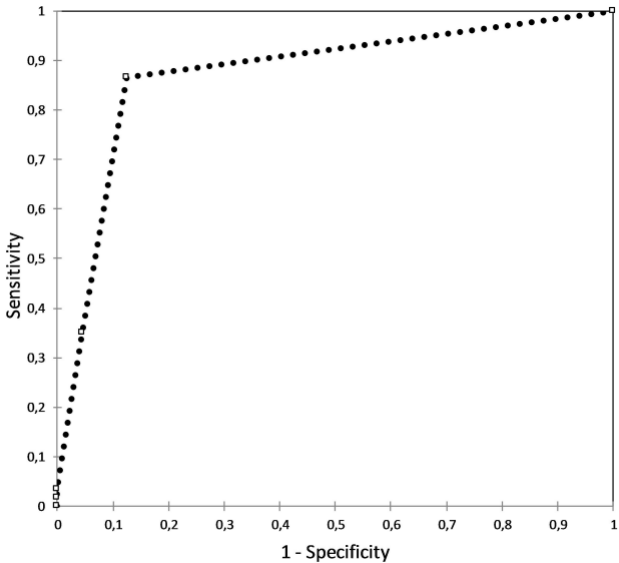

Supplement: Supplementary file 2 [file IJLH-41-782-s002.pdf]

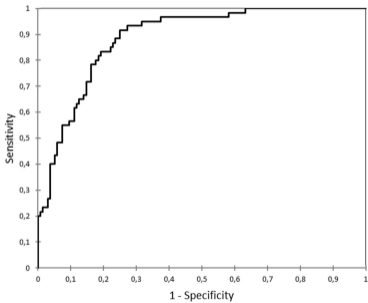

Supplement: Supplementary file 3 [file IJLH-41-782-s003.pdf]

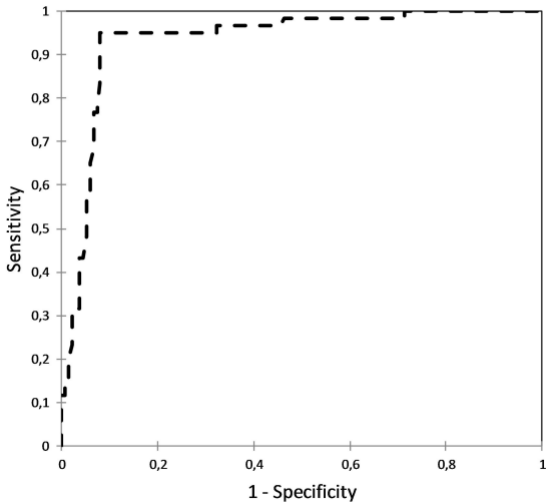

Supplement: Supplementary file 4 [file IJLH-41-782-s004.pdf]
