# Supplementary material for: A hierarchical approach in the diagnostic workflow of chronic myelomonocytic leukemia: Pivotal role of the “Mono‐dysplasia‐score” combined with flow cytometric quantification of monocyte subsets
Source: Int J Lab Hematol. 2019 Oct 24;41(6):782–90. doi: 10.1111/ijlh.13115 (PMC6916376; doi:10.1111/ijlh.13115)
Supplement: Supplementary file 5 [file IJLH-41-782-s005.docx]

**Supplemental material**

Supplementary Table 1. Scoring of dysplasia on blood smear examination.

| **Cell dysplasia** | **<10%** | **≥ 10%** |
| --- | --- | --- |
| Hypogranulated neutrophils | 0 | 1 |
| Hyposegmented neutrophils |  |  |
| Abnormal monocytes |  |  |
| Giant platelets/ Macrothrombocytes |  |  |

Supplementary Table 2. Details of antibody clones and fluorochromes used for flow cytometry

| **Center** | **CD14** | | **CD16** | |
| --- | --- | --- | --- | --- |
|  | **Fluorochrome** | **Clone** | **Fluorochrome** | **Clone** |
| Besançon | APC-H7 | MɸP9 | FITC | 3G8 |
| Caen | APC-H7 | MɸP9 | PE | B73.1 |
| Paris | PE-CY7 | RMO52 | Pacific Blue | 3G8 |

Supplementary Figure 1.

Description of the study population. 271 patients falling within the WHO criteria (monocyte count ≥ 1 10^9^/L and accounting for ≥ 10% of the total WBC) were included in this multicentric study. Patients younger than age 50 (n= 35), with overt context of non-clonal monocytosis (active sepsis, n= 25), ineligible to flow cytometry (sample > 24h, recovery from aplasia, n= 11) or without follow-up (n=4) were excluded, leaving 196 samples for analysis.

Supplementary Figure 2.

1. ROC curve for blood smear dysplasia score. AUC=0.88, 95% CI = (0.83 -0.93). With a threshold of 1, sensitivity and specificity are 87%, 95%CI = (76% - 93%) and 88%, 95% CI= (82% - 93%) respectively.
2. ROC curve for Monoscore. AUC= 0.88, 95% CI = (0.82 -0.93). With a threshold of 0.161, sensitivity and specificity are 93%, 95%CI = (84% - 98%) and 51%, 95% CI= (42% - 59%) respectively.
3. ROC curve for classical monocytes fraction. AUC= 0.93, 95% CI = (0.89 -0.98). With a threshold of 94%, sensitivity and specificity are 93%, 95%CI = (84% - 98%) and 83%, 95% CI= (76% - 89%) respectively.
